# Carbenoxolone Blocks the Light-Evoked Rise in Intracellular Calcium in Isolated Melanopsin Ganglion Cell Photoreceptors

**DOI:** 10.1371/journal.pone.0022721

**Published:** 2011-07-29

**Authors:** Jayne R. Bramley, Erin M. Wiles, Patricia J. Sollars, Gary E. Pickard

**Affiliations:** School of Veterinary Medicine and Biomedical Sciences, University of Nebraska, Lincoln, Nebraska, United States of America; University of California, Berkeley, United States of America

## Abstract

**Background:**

Retinal ganglion cells expressing the photopigment melanopsin are intrinsically photosensitive (ipRGCs). These ganglion cell photoreceptors send axons to several central targets involved in a variety of functions. Within the retina ipRGCs provide excitatory drive to dopaminergic amacrine cells via glutamatergic signals and ipRGCs are coupled to wide-field GABAergic amacrine cells via gap junctions. However, the extent to which ipRGCs are coupled to other retinal neurons in the ganglion cell layer via gap junctions is unclear. Carbenoxolone, a widely employed gap junction inhibitor, greatly reduces the number of retinal neurons exhibiting non-rod, non-cone mediated light-evoked Ca^2+^ signals suggesting extensive intercellular coupling between ipRGCs and non-ipRGCs in the ganglion cell layer. However, carbenoxolone may directly inhibit light-evoked Ca^2+^ signals in ipRGCs independent of gap junction blockade.

**Methodology/Principal Findings:**

To test the possibility that carbenoxolone directly inhibits light-evoked Ca^2+^ responses in ipRGCs, the light-evoked rise in intracellular Ca^2+^ ([Ca^2+^]_i_) was examined using fura-2 imaging in isolated rat ipRGCs maintained in short-term culture in the absence and presence of carbenoxolone. Carbenoxolone at 50 and 100 µM concentrations completely abolished the light-evoked rise in [Ca^2+^]_i_ in isolated ipRGCs. Recovery from carbenoxolone inhibition was variable.

**Conclusions/Significance:**

We demonstrate that the light-evoked rise in [Ca^2+^]_i_ in isolated mammalian ganglion cell photoreceptors is inhibited by carbenoxolone. Since the light-evoked increase in [Ca^2+^]_i_ in isolated ipRGCs is almost entirely due to Ca^2+^ entry via L-type voltage-gated calcium channels and carbenoxolone does not inhibit light-evoked action potential firing in ipRGCs *in situ*, carbenoxolone may block the light-evoked increase in [Ca^2+^]_i_ in ipRGCs by blocking L-type voltage-gated Ca^2+^ channels. The ability of carbenoxolone to block evoked Ca^2+^ responses must be taken into account when interpreting the effects of this pharmacological agent on retinal or other neuronal circuits, particularly if a change in [Ca^2+^]_i_ is the output being measured.

## Introduction

Retinal ganglion cells expressing melanopsin are intrinsically photosensitive (ipRGCs) [1], [2] and send signals to a variety of central targets involved in visual perception and non-image forming functions [2]–[8]. Within the retina, ipRGCs provide light-evoked excitatory (glutamatergic) drive to a subset of dopaminergic amacrine cells that play a key role in reconfiguring retinal function according to prevailing illumination conditions [9]. Signals from ipRGCs may also propagate to other neurons in the ganglion cell layer of the retina, such as wide-field GABAergic amacrine cells, via electrical coupling through gap junctions [10].

Gap junctions/electrical synapses are abundant throughout the plexiform layers of the retina and are diverse in their ultrastructural morphology [11], [12]. Electrical coupling via gap junctions plays an important role in communication among many types of retinal neuron [11], [13]. Extensive gap junction-mediated coupling between ipRGCs and other retinal neurons in the ganglion cell layer was first reported by Sekaran and colleagues [14], [15] based on the large reduction (>50%) of retinal cells exhibiting light-evoked increases in cytosolic Ca^2+^ in the presence of carbenoxolone, a compound widely used to block gap junctions [16]. However, carbenoxolone similarly applied to the retina maintained *in vitro* for multielectrode recording does not decrease the number of retinal neurons generating light-evoked action potentials [17], [18]. The explanation for these divergent results is currently unknown but may be related to the different endpoints that were measured in these studies: light-evoked action potentials [17], [18] vs light-evoked calcium responses [14], [15].

The primary cellular mechanism mediating the light-evoked elevation in ipRGC intracellular calcium levels ([Ca^2+^]_i_) is by Ca^2+^ influx through L-type voltage-gated calcium channels (VGCC) (Cav1 of more recent nomenclature [19]); ≈90% of the light-evoked increase in ipRGC [Ca^2+^]_i_ in isolated cells *in vitro* was attributed to L-type VGCC activation subsequent to light-evoked depolarization and action potential firing [20]. Thus, if in addition to blocking gap junctions, carbenoxolone also acts downstream of light-evoked depolarization and action potential generation to inhibit L-type VGCC, then carbenoxolone would inhibit the light-evoked elevation in [Ca^2+^]_i_ while having no measurable effect on light-evoked action potential firing in ipRGCs. Indeed, carbenoxolone does suppress Ca^2+^ signals in isolated amphibian cone photoreceptors and reduces depolarization-evoked [Ca^2+^]_i_ responses in amphibian retinal slices by blocking voltage-gated calcium channels [21]. It is not known if carbenoxolone acts directly on mammalian ganglion cell photoreceptors to inhibit light-evoked Ca^2+^ responses.

In this study we examined light-evoked calcium responses of isolated ipRGCs maintained *in vitro* in the absence and presence of carbenoxolone. Carbenoxolone blocked completely the light-evoked increase in [Ca^2+^]_i_ in isolated ipRGCs. The data indicate that evaluation of gap junction coupling using carbenoxolone as a blocker and changes in [Ca^2+^]_i_ as an output measure must consider a possible direct effect of this compound on membrane calcium channels.

## Results

### Light-evoked Ca^2+^ response in isolated ipRGCs

**Figure 1:**
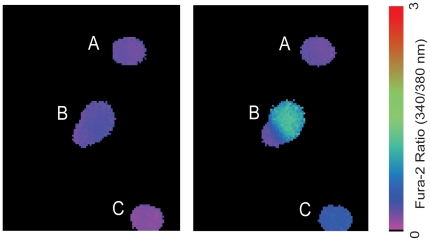
Pseudocolored images of fura-2 fluorescence ratios (340/380 nm) for melanopsin-panned retinal ganglion cells before and during light stimulation. Cells labeled B and C responded to the broad-spectrum 60 sec light pulse with an increased [Ca^2+^]_i_ establishing these cells as ipRGCs. The cell labeled A was unresponsive to the light pulse and the baseline [Ca^2+^]_i_ did not change.

Calcium imaging experiments were conducted on cultured melanopsin-expressing ipRGCs 1–2 days after their isolation from neonatal rats. Retinal ganglion cells isolated by melanopsin-immunopanning were cultured at low density allowing analyses to be performed on individual cells that were not in physical contact with other ipRGCs. Melanopsin-immunopanned RGCs retained their intrinsic photosensitivity *in vitro* and responded to light stimuli with an elevation in [Ca^2+^]_i_ that rapidly returned toward baseline levels after termination of the light stimulus (Fig. 1).

### The effect of carbenoxolone on the light-evoked Ca^2+^ response in ipRGCs

**Figure 2:**
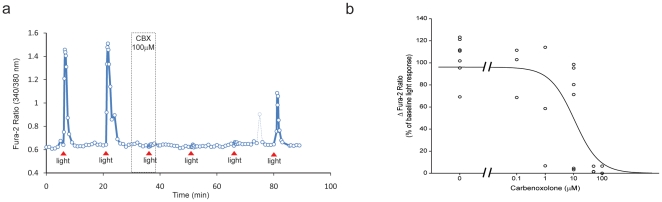
Light-evoked Ca^2+^ responses in ipRGCs in the absence and presence of carbenoxolone. (a) The light-evoked elevation in [Ca^2+^]_i_ in an isolated ipRGC was completely blocked in the presence of 100 µM carbenoxolone (CBX) with only partial recovery after the third light pulse presented after 45 min of wash out. (b) Dose-response data illustrating inhibition of baseline light-evoked Ca^2+^ response in the presence of 0.1, 1, 10, 50 and 100 µM carbenoxolone. Only ipRGCs showing at least partial recovery after carbenoxolone washout are included. The light-evoked Ca^2+^ responses from seven ipRGCs in the absence of carbenoxolone (0 µM) are included. These cells gave four consecutive light-evoked Ca^2+^ responses (values provided are the third light-evoked Ca^2+^ response compared to preceding light-evoked Ca^2+^ response as baseline). The curve was fit with a three-parameter logistic equation (EC_50_ = 11 µM). Non-evoked elevations in [Ca^2+^]_i_ in isolated ipRGCs, due to spontaneous action potential firing, are de-emphasized by plotting data with lightened dashed lines.

To determine the direct effect of the gap junction blocker carbenoxolone on light-induced Ca^2+^ responses in individual ipRGCs, carbenoxolone was delivered to the recording chamber after two light-evoked Ca^2+^ responses had been recorded. Carbenoxolone reduced the light-evoked Ca^2+^ response in isolated ipRGCs in a concentration-related manner (0.1–100 µM) although the extent of inhibition was quite variable among ipRGCs at the intermediate concentrations tested (1 and 10 µM) and complete inhibition of the light-evoked Ca^2+^ response was observed in at least some cells at all concentrations examined except 100 nM. In the presence of 100 µM carbenoxolone, the concentration typically used to block gap junctions between neurons maintained *in vitro* [14], [21]–[25], the light-induced Ca^2+^ response in individual ipRGCs was completely abolished (1.6±1.2% of the baseline light-evoked Ca^2+^ response; n = 5, p<0.01) (Figs. 2a and 2b).

Recovery from carbenoxolone inhibition of the light-evoked Ca^2+^ response in individual ipRGCs was evaluated by recording three light-evoked Ca^2+^ responses spaced 15 min apart during carbenoxolone washout. The level of recovery of the light-evoked Ca^2+^ response was related to the concentration of carbenoxolone tested with greater recovery at the lower concentrations. However, recovery of the light-evoked Ca^2+^ response was variable; 5 of 20 cells showed complete recovery whereas 8 of the 20 ipRGCs showed no recovery after 45 min (light-evoked Ca^2+^ response <5% of the base line light-evoked Ca^2+^ response, Table 1). These data are in agreement with findings reported by others indicating that it is difficult or impossible to reverse the effects of carbenoxolone [26].

**Table 1:** Carbenoxolone inhibition of the light-evoked Ca^2+^ response in solitary ipRGCs in vitro is difficult to reverse.

Carbenoxolone Dose (µM)	Recovery of Light-evoked Ca^2+^ Response	ipRGC (n)
100	complete recovery (30 min)	1
	partial recovery	1
	no recovery	3
		
50	complete recovery (15 min)	2
	partial recovery	1
	no recovery	1
		
10	no inhibition	2
	complete recovery (15 min)	1
	partial recovery	3
	no recovery	1
		
1.0	no inhibition	1
	complete recovery (15 min)	1
	partial recovery	1
	no recovery	3
		
0.1	no inhibition	2
	partial recovery	1

Complete Recovery: ipRGCs demonstrating light-evoked Ca^2+^ responses during carbenoxolone (CBX) washout (45 min) that were ≥100% of the light-evoked Ca^2+^ response immediately prior to drug treatment were classified as demonstrating complete recovery (n = 5). (time elapsed when complete recovery was noted)

Partial Recovery: ipRGCs demonstrating light-evoked Ca^2+^ responses during CBX washout (45 min) that were greater than the light-evoked Ca^2+^ response during CBX application and between 50–90% of the light-evoked Ca^2+^ response immediately prior to drug treatment were classified as demonstrating partial recovery (n = 7).

No Recovery: ipRGCs demonstrating light-evoked Ca^2+^ responses during CBX washout (45 min) that remained <5% of the light-evoked Ca^2+^ response immediately prior to drug treatment were classified as demonstrating no recovery (n = 8).

No Inhibition: ipRGCs demonstrating light-evoked Ca^2+^ responses in the presence of CBX that were between 95–120% of the preceding light-evoked Ca^2+^ response in the absence of CBX were classified as demonstrating no inhibition (n = 5).

### Glutamate-evoked Ca^2+^ responses in isolated ipRGCs

**Figure 3:**
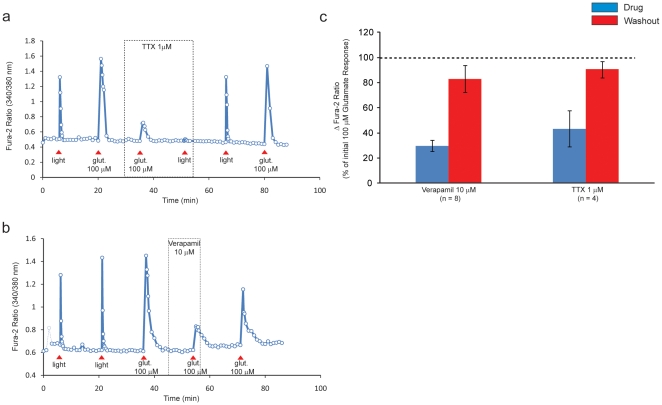
Glutamate-evoked Ca^2+^ responses in ipRGCs in the absence and presence of tetrodotoxin or verapamil. (a) The glutamate-evoked (Glut 100 µM) Ca^2+^ response in an isolated ipRGC was reversibly inhibited by tetrodotoxin (TTX, 1 µM) and the light-evoked rise in [Ca^2+^]_i_ was completely blocked. (b) The glutamate-evoked increase in [Ca^2+^]_i_ was reversibly inhibited by verapamil (10 µM). (c) Summary (means ± SEM, normalized to the pretreatment responses) demonstrating reversible inhibition of the glutamate-evoked rise in [Ca^2+^]_i_ by tetrodotoxin (n = 4) and verapamil (n = 8). p<0.01, one-way repeated measures ANOVA, Tukey's post-hoc test compared to initial and recovery responses for TTX and verapamil.

In an attempt to demonstrate that ipRGCs remained capable of generating an evoked Ca^2+^ response after 100 µM carbenoxolone application and washout despite the lack of a light-evoked Ca^2+^ response, we examined glutamate-evoked Ca^2+^ responses; it has been shown previously that glutamate application (10–100 µM) evokes a rise in [Ca^2+^]_i_ in isolated ipRGCs [20]. The glutamate-evoked rise in [Ca^2+^]_i_ in ipRGCs could result from Ca^2+^ admitted via NMDA and/or AMPA/kainate-type glutamate receptors and via the activation of VGCC occurring after membrane depolarization and action potential firing resulting from glutamate receptor stimulation, as described for immunopanned conventional RGCs [27]. We first confirmed that brief glutamate application (100 µM; 15 sec) evoked action potentials in isolated ipRGCs by stimulating cells with glutamate in the absence and presence of tetrodotoxin (TTX) which prevents action potential generation by blocking voltage-gated Na^+^ channels. TTX (1 µM) significantly inhibited the glutamate-evoked rise in [Ca^2+^]_i_ with responses 43.5±10.5% of baseline (n = 4, p<0.01) (Fig. 3a,c). In addition, TTX produced a robust inhibition of the light-evoked increase in [Ca^2+^]_i_ in all cells tested (Fig. 3a), confirming previous observations [20]. These data suggest that the glutamate-evoked rise in [Ca^2+^]_i_ resulted from Ca^2+^ entry via glutamate receptors permeant to Ca^2+^ and with the majority of the elevation in [Ca^2+^]_i_ due to activation of VGCC following action potential generation.

To confirm that VGCC are activated by glutamate in isolated ipRGCs we stimulated cells with glutamate (100 µM; 15 sec) in the absence and presence of verapamil, an L-type VGCC antagonist, that has been shown previously to inhibit light-evoked activation of L-type VGCC in isolated ipRGCs resulting from action potential generation [20]. Verapamil (10 µM) significantly reduced the glutamate-evoked elevation in [Ca^2+^]_i_ (29.7±4.4% of baseline response, n = 8, p<0.01) (Fig. 3b,c). These data and the TTX data described above suggest that the glutamate-evoked rise in [Ca^2+^]_i_ results from Ca^2+^ admitted through glutamate receptors followed by action potential generation and activation of L-type VGCC.

**Figure 4:**
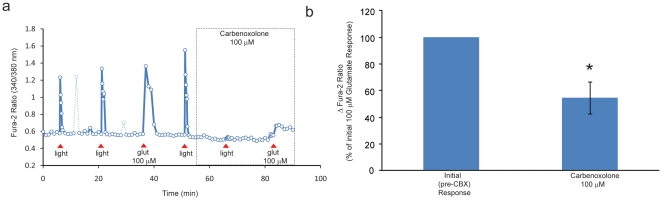
Carbenoxolone inhibition of glutamate-evoked Ca^2+^ responses in ipRGCs. (a) Carbenoxolone (CBX, 100 µM) completely blocked the light-evoked rise in [Ca^2+^]_i_ and inhibited the glutamate-evoked elevation in [Ca^2+^]_i_ (b) Summary (means ± SEM, normalized to the pretreatment responses) illustrating the carbenoxolone inhibition of glutamate-evoked rise in Ca^2+^ (following complete inhibition of the light-evoked Ca^2+^ response). * p<0.01, Student's t-test (n = 7)

Lastly we asked whether the glutamate-evoked rise in [Ca^2+^]_i_ is reduced by carbenoxolone. After establishing that cells were light responsive and that they responded to glutamate (100 µM; 15 sec) with an evoked rise in [Ca^2+^]_i_ (baseline response), cells were stimulated with light in the absence of carbenoxolone (for a baseline Ca^2+^ response) followed by light in the presence of carbenoxolone (100 µM) and then glutamate (100 µM; 15 sec) in the presence of carbenoxolone. The light-evoked Ca^2+^ response was again completely blocked by 100 µM carbenoxolone, (3.0±0.4% of baseline response, n = 7, p<0.001, Student's t-test) (Figs. 4a,b) confirming the findings in the initial experiment described above. Carbenoxolone also significantly reduced the glutamate-evoked elevation in [Ca^2+^]_i_ (54.7±12.1% of baseline, n = 7, p<0.01) (Figs. 4a,b).

These latter data indicate that in the presence of 100 µM carbenoxolone, ipRGCs remained viable and were capable of generating a glutamate-evoked Ca^2+^ response despite their lack of an increase in [Ca^2+^]_i_ in response to light stimulation. This finding validates the observed inhibition of the light-evoked Ca^2+^ response in the absence of recovery from carbenoxolone in ipRGCs in the initial experiment above. Comparison of the data from the initial experiment of 100 µM carbenoxolone inhibition of light-evoked Ca^2+^ responses (n = 5) with this last experiment examining 100 µM carbenoxolone inhibition of light-evoked Ca^2+^ responses (n = 7) revealed no significant difference in the level of carbenoxolone inhibition (1.6±1.2% vs 3.0±0.4%, p>0.05). It can be concluded that 100 µM carbenoxolone virtually eliminates the light-evoked elevation in [Ca^2+^]_i_ in isolated ipRGCs. In addition, comparison of the glutamate-evoked Ca^2+^ responses in the presence of TTX, verapamil, or carbenoxolone revealed no significant difference in the inhibitory effect of the three compounds on the level of the glutamate-evoked elevation of [Ca^2+^]_i_ (one-way ANOVA, p>0.10).

## Discussion

The principal novel finding in this study is the demonstration that carbenoxolone, a widely used agent for inhibiting neuronal gap junctions, abolished the light-evoked elevation in [Ca^2+^]_i_ in solitary, isolated mammalian retinal ganglion cell photoreceptors maintained in short-term culture. Importantly, carbenoxolone blocked the light-evoked Ca^2+^ response by acting directly on the ipRGCs as these neurons lacked any physical contact with other ipRGCs or other retinal cells in culture. The effects of carbenoxolone on evoked-Ca^2+^ responses in the present study were obtained by stimulating isolated, individual mammalian retinal neuron photoreceptors with light, coupled with non-invasive calcium imaging. Our findings are in agreement with those of Vessey and colleagues [21] who examined the effects of carbenoxolone on calcium currents (with Ba^2+^ as a charge carrier) obtained by depolarizing voltage-clamped amphibian cone photoreceptors using whole-cell patch-clamp procedures. The data are consistent with the interpretation that carbenoxolone directly blocks L-type voltage-gated calcium channels. Although the possibility that carbenoxolone may have stimulated the release of unknown tropic factors from cells in the cultures that subsequently inhibited the evoked Ca^2+^ responses can not be entirely eliminated, it is clear that the effect of carbenoxolone on the light-evoked Ca^2+^ response in the present study was not the result of carbenoxolone blocking gap junctions between ganglion cell photoreceptors.

It was first reported that ipRGCs were extensively coupled to other cells in the ganglion cell layer of the retina via gap junctions based on the reduction in the number of cells demonstrating a light-evoked Ca^2+^ response in the presence of carbenoxolone [14], [15]. Sekaran and colleagues measured light-evoked Ca^2+^ responses in ipRGCs using fura-2 AM ratiometric imaging in the absence and presence of 100 µM carbenoxolone [14], similar to the procedures used in the current study, but with an important difference. In the current study light-evoked Ca^2+^ responses were measured in individual ipRGCs in isolation, whereas Sekaran and co-workers examined light-evoked Ca^2+^ responses in retinas maintained *in vitro* [14]. It is clear from the present study, that carbenoxolone applied to retinas *in vitro* [14], [15] would have inhibited the light-evoked elevation in [Ca^2+^]_i_ in ipRGCs, in addition to blocking gap junction coupling between retinal cells. This direct action of carbenoxolone on light-evoked Ca^2+^ responses in ipRGCs would have resulted in Sekaran and colleagues greatly overestimating the extent of coupling between ipRGCs and other cells in the ganglion cell layer of the retina [14], [15]. It has recently been shown that ipRGCs in the mouse retina injected with the tracer Neurobiotin are tracer-coupled to wide-field GABAergic amacrine cells in the ganglion cell layer; there was no evidence for homologous tracer-coupling of ipRGCs or heterologous coupling to other types of ganglion cells [10]. Thus, it is possible that following the light-evoked depolarization of ipRGCs, calcium signals could propagate from ipRGCs to displaced GABAergic amacrine cells via gap junctions, explaining the carbenoxolone-induced reduction in measurable light-evoked Ca^2+^ responses in the ganglion cell layer reported by Sekaran and colleagues [14], [15]. However, a more likely explanation for the large reduction (rather than complete elimination) of the light-evoked Ca^2+^ responses in cells in the ganglion cell layer described by Sekaran and co-workers [14], is that the carbenoxolone blockade of light-evoked Ca^2+^ responses in ipRGCs in the intact retina was simply incomplete due to diffusion barriers [14], [15].

The interpretation that the inhibitory effect of carbenoxolone on the light-evoked Ca^2+^ response in ipRGCs was due to the blockade of L-type VGCC is based on several observations. The primary source for light-evoked elevations in ipRGC [Ca^2+^]_i_ is via L-type VGCC after light-induced depolarization and action potential firing [20]; Vessey and colleagues [21] reported carbenoxolone inhibition of VGCC in amphibian cone photoreceptors. However, the light-evoked rise in Ca^2+^ in ipRGCs occurs after photon capture by melanopsin and membrane depolarization which is believed to be mediated via canonical transient receptor potential (TRP) channels [20], [28]–[30]. If in our study carbenoxolone blocked TRP C channels then it would have inhibited the light-induced rise in Ca^2+^ by preventing the ipRGCs from depolarizing and firing action potentials. However, carbenoxolone did not alter light-evoked action potential generation in ipRGCs [17], [18] making it very unlikely that in our study carbenoxolone inhibited TRP C channels in ipRGCs to any significant extent or effected light-induced action potential generation.

Additional evidence in support of the interpretation that carbenoxolone blocks L-type VGCC comes from the glutamate-evoked elevation in [Ca^2+^]_i_ in ipRGCs described in this study. Glutamate acting via glutamate receptors on ipRGCs produces membrane depolarization followed by action potential generation and activation of L-type VGCC; the glutamate evoked rise in Ca^2+^ was significantly reduced by TTX which blocks action potential generation. The residual Ca^2+^ response in the presence of TTX was from Ca^2+^ entry via NMDA and/or AMPA/kainate-type glutamate receptors which are permeable to Ca^2+^. Thus, blocking glutamate-evoked action potentials eliminates activation of L-type VGCC. Indeed, we next demonstrated that verapamil, a selective L-type VGCC antagonist, inhibited the glutamate-evoked rise in Ca^2+^ to the same degree as blockade by TTX. Thus if carbenoxolone blocked L-type VGCC, it would be predicted that carbenoxolone would produce a similar level of antagonism as TTX and verapamil; the carbenoxolone inhibition of the glutamate-evoked elevation in [Ca^2+^]_i_ in ipRGCs was not significantly different from that observed with TTX or verapamil. Taken together, the data strongly suggest that carbenoxolone inhibits L-type VGCC.

Although carbenoxolone is a widely used gap junction inhibitor, the effects of carbenoxolone are difficult and at times impossible to reverse, as reported here and as described in the literature. Pharmacological agents that block gap junctions and are potentially more easily reversible, such as the fenamates, have been sought [31]. Flufenamic acid derivatives have been shown to block gap junction coupling between neurons and meclofenamic acid is a very effective gap junction blocker in the retina [26], [32], [33]. Importantly, meclofenamic acid has also been reported not to reduce the number of retinal ganglion cells (ipRGCs) exhibiting light-evoked action potentials, similar to carbenoxolone application [18]. However, also similar to carbenoxolone, in the presence of meclofenamic acid (100 µM), light-evoked increases in [Ca^2+^]i were completely blocked in individual immunopanned ipRGCs, and the effect was not easily reversible (Bramley, Sollars and Pickard, unpublished observations). Thus, inhibition of VGCC should also be considered when using meclofenamic acid as a gap junction blocker.

In summary, carbenoxolone, a water-soluble membrane permeant pharmacological agent derived from licorice root, inhibits gap junctions but also has actions on other membrane channels including VGCC. Thus it is not a compound that can be used reliably when Ca^2+^ responses are the outcome measure or when attempting to assign a role for gap junctions in complex physiological network responses such as those that occur in the retina.

## Materials and Methods

### Ethics Statement

The use of animals in this study was approved by the University of Nebraska-Lincoln Institutional Animal Care and Use Committee (ID 391) and all experiments were conducted according to NIH guidelines in Principles of Laboratory Animal Care.

### Immunopanned RGC Cultures

Isolation of melanopsin-expressing ipRGCs was performed as previously described [20]. Briefly, Long-Evans rat pups (6-8 per panning session) were killed at age 5–7 days postnatal by halothane overexposure and decapitation. A single-cell retinal suspension was produced through enzymatic dissociation of dissected retinas (in Ca^2+^/Mg^2+^-free Dulbecco's phosphate-buffered saline [DPBS] with papain and DNase) and mechanical trituration; melanopsin-expressing RGCs were isolated using a melanopsin-antibody panning protocol with a rabbit melanopsin antibody (F7884) generated against the N-terminal 19 amino acid sequence of rat melanopsin. Melanopsin-panned cells were plated into wells containing a poly-D-lysine/laminin-coated Biocoat glass coverslip and 750 µl of culture medium consisting of Neurobasal-A plus 2% B27 supplements, 1 mM glutamine, 25 ng/ml BDNF, 10 ng/ml CNTF, 5 µM forskolin, and 10 µg/ml gentamicin. Cultures were maintained in the dark at 37°C in a humidified 5% CO_2_-air atmosphere.

### Calcium Imaging

After 1–2 days in culture, the coverslip-plated melanopsin-panned cells were transferred to modified Hank's balanced salt solution (HBSS; 15 mM HEPES, 2.6 mM CaCl2, Mg^2+^-free, pH 7.4) containing 5 µM fura-2 AM calcium indicator dye for 30 min in the dark at 35°C. The fura-loaded cells were then transferred to a microscope chamber that was constantly superfused with HBSS warmed to 33–35°C and bubbled with 100% oxygen. All tested drugs were dissolved in the HBSS and delivered to the chamber by a peristaltic pump at a rate of ≈1 ml/min.

For calcium imaging, a 100 W mercury lamp and the appropriate filters (excitation 340 or 380 nm; emission 510 nm) were used to generate fura-2 fluorescence. A filter wheel alternated the 340 and 380 nm filters with a 400 ms excitation period for each wavelength. Fluorescence images (12-bit) were captured with a cooled charged-coupled device camera fitted to an Axioskop 2 FS upright microscope using a water-immersion objective (N.A. 0.80W; Achroplan 40x). The images of RGC fluorescence at 340 and 380 nm excitation were calculated over a large area of the RGC soma and converted to ratiometric (340/380 nm) images by imaging software (Imaging Workbench 2.2; Axon Instruments, Foster City, CA). The background fluorescence was measured from a region on the coverslip devoid of RGCs and subtracted from each image.

To minimize the intensity of the fura-2 excitation wavelengths (and reduce potential ipRGC stimulation), 8 X 8 pixel binned images were acquired. With this increased binning, adequate fluorescence was attained after reducing the 340 and 380 nm excitation intensities with −0.5 and −2.5 log neutral density filters, respectively. Light responses were calculated as the Δ fura-2 ratio, the peak minus baseline fura-2 ratio. The baseline response was calculated as the average fura-2 ratio of the 3 ratios acquired prior to light or drug exposure and the peak was the maximum fura-2 ratio observed in the 100 s following light offset or drug termination.

The isolated cells were stimulated with broad-spectrum light from a 100 W halogen bulb, with light passing through the condenser underneath the microscope chamber. One minute light stimuli were used and the halogen light was blocked every 10 s to allow for the acquisition of fura-2 images. Light stimulation was remotely triggered using an electronic shutter. The standard light stimulus utilized in the experiments was measured as approximately 2.0×10^13^ photons/s/cm at 420 nm, 6.9×10^13^ photons/s/cm at 480 nm, 1.7×10^14^ photons/s/cm at 540 nm and 3.3×10^14^ photons/s/cm at 600 nm.
